# Acquired hemophilia A developing in the perioperative period of pancreatoduodenectomy: a report of two cases

**DOI:** 10.1186/s40792-023-01656-1

**Published:** 2023-05-10

**Authors:** Hidemasa Kubo, Ryo Ashida, Katsuhisa Ohgi, Masafumi Fukaya, Naoki Umezaki, Mihoko Yamada, Shimpei Otsuka, Katsuhiko Uesaka, Teiichi Sugiura

**Affiliations:** 1 Division of Hepato-Biliary-Pancreatic Surgery, Shizuoka Cancer Center, 1007 Shimonagakubo, Sunto-Nagaizumi, Shizuoka, 4118777 Japan; 2 Division of Hematology and Stem Cell Transplantation, Shizuoka Cancer Center, 1007 Shimonagakubo, Sunto-Nagaizumi, Shizuoka, 4118777 Japan

**Keywords:** Acquired hemophilia A, Pancreatoduodenectomy, Perioperative period

## Abstract

**Background:**

Acquired hemophilia A (AHA) is a rare disease characterized by a prolonged activated partial thromboplastin time (aPTT) and the production of coagulation factor VIII inhibitors. We encountered two cases of AHA in the perioperative period of pancreatoduodenectomy (PD).

**Case presentation:**

*Case 1*: A 76-year-old woman with intraductal papillary mucinous carcinoma developed acute cholecystitis 5 days before PD. Despite immediate improvement in her acute cholecystitis with biliary drainage and antibiotics, her aPTT level was prolonged (55.9 s). PD was performed as scheduled. On postoperative day (POD) 2, she developed intra-abdominal hemorrhaging that required reoperation. However, intra-abdominal bleeding and concomitant anemia persisted after reoperation. On POD 13, she was diagnosed with AHA based on the detection of an inhibitor of coagulation factor VIII. Despite hemostatic and immunosuppressive treatment, including massive blood transfusion, her general condition gradually worsened due to continuous bleeding and secondary infections. She ultimately died of multiple organ failure on POD 71. *Case 2*: An 82-year-old man received PD for distal cholangiocarcinoma. On POD 3, a small amount of blood via abdominal drainage was observed. On POD 4, his aPTT level was prolonged (61.5 s). On POD 8, subcutaneous hemorrhaging of the median wound was observed, and corticosteroids were administered under suspicion of AHA on POD 9. On POD 15, an inhibitor of FVIII was detected, and he was diagnosed with AHA. On POD 17, the aPTT level had normalized, and an inhibitor of FVIII was undetectable. On POD 41, he was discharged without any serious hemorrhagic events.

**Conclusions:**

AHA may be more frequent than previously reported. When unexplained prolonged aPTT or bleeding symptoms are observed, it is important to keep AHA in mind during the perioperative period of invasive surgery.

## Introduction

Acquired hemophilia A (AHA) is a rare autoimmune disease characterized by an unjustified prolonged activated partial thromboplastin time (aPTT) and abnormal acute bleeding symptoms with no personal or family history of coagulopathy—due to the production of inhibitor of coagulation factor VIII (FVIII)—with a normal prothrombin time (PT) [1]. The incidence of AHA has been reported to range from 1 to 6 cases per million population per year [1]. Because of its rare incidence, it has been pointed out that a delay in the diagnosis and appropriate treatment commonly puts patients at unnecessary risk of severe bleeding [1, 2]. Although the reported incidence has gradually increased in recent years, as AHA has become recognized [3, 4], the condition is not yet recognized widely enough.

We encountered two cases of AHA in just 2 years and herein report those cases that developed during the perioperative period of pancreatoduodenectomy (PD), following markedly different clinical courses.

## Case presentation

### Case 1

**Fig. 1:**
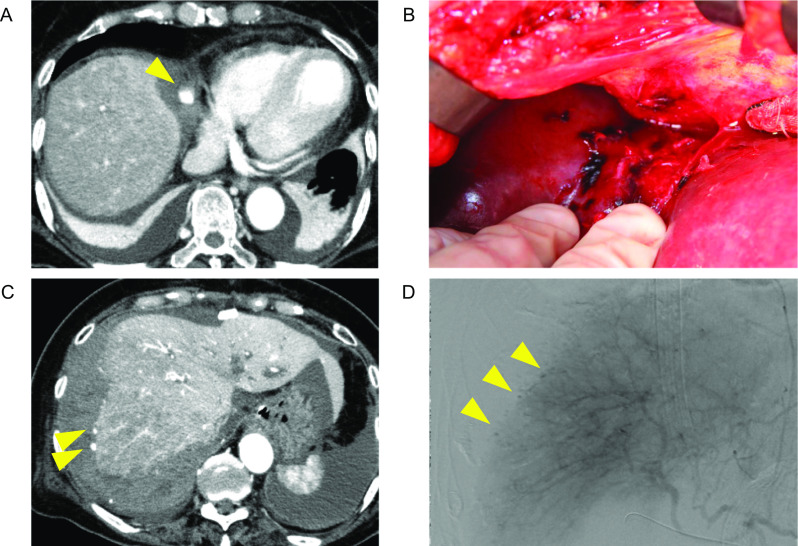
Findings of Case 1. **A** CE-CT (arterial phase) on POD2. The yellow arrowhead shows the extravasation of contrast medium from the hepatic falciform ligament artery. **B** Intraoperative findings of hemostatic surgery on POD2. **C** CE-CT (arterial phase) on POD 8. Yellow arrowheads show the extravasation of contrast medium from the liver surface. **D** The findings of angiography of the hepatic artery on POD8. Yellow arrowheads show the extravasation of contrast medium from the liver surface. CE-CT: contrast-enhanced computed tomography; POD: postoperative day

**Fig. 2:**
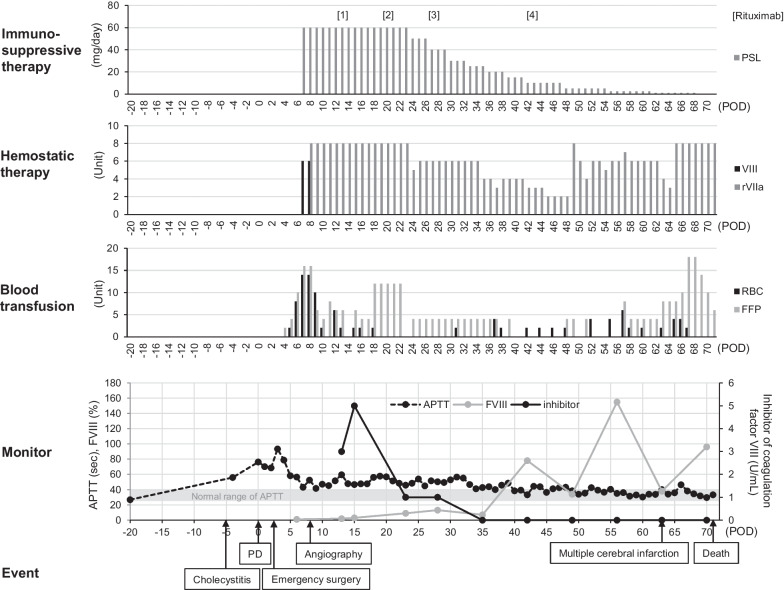
Detailed clinical course of Case 1. The top row shows the dose of immunosuppressive therapy (rituximab and prednisolone). The second row shows the dose of hemostatic therapy with the FVIII agent and rFVIIa. The third row shows the details of blood transfusion of RBCs and FFPs. The fourth row shows the clinical course of aPTT, FVIII activity and the inhibitor of FVIII. The bottom row shows the clinical events. aPTT: activated partial thromboplastin time; FFP: fresh frozen plasma; FVIII: coagulation factor VIII; POD: postoperative day; PSL: prednisolone; RBC: red blood cells

A 76-year-old woman was scheduled for pancreatoduodenectomy (PD) for intraductal papillary mucinous carcinoma of the pancreas head. The aPTT level at the time of the initial visit was 26.8 s (normal range at the institute: 25.0–40.0 s). She developed acute cholecystitis 5 days prior to elective surgery because of biliary congestion caused by the tumor. Acute cholecystitis was immediately improved with biliary drainage and the administration of antibacterial agents. However, her aPTT level was prolonged to 55.9 s after cholecystitis, whereas the prothrombin time (PT) was within the normal range (13.0 s [normal range at the institute: 10.5–13.5 s]). Prolonged aPTT was considered due to infection or bacteremia, and she had no clinical bleeding tendency. PD was performed as scheduled without further investigation. She received a transfusion of 4 units of red blood cells (RBCs) for intraoperative blood loss (a total of 1614 g) due to prolonged oozing from the operative field. On postoperative day (POD) 1, the amylase level of her abdominal drainage fluid was 45 U/L (upper limit of the serum amylase level at the institute: 125 U/L). On POD 2, she had intra-abdominal hemorrhaging, and contrast-enhanced computed tomography (CE-CT) showed extravasation from the hepatic falciform ligament artery (Fig. 1A). She subsequently underwent reoperation that identified hematoma around the liver and arterial hemorrhaging from the hepatic falciform ligament. Hemostasis by ligation and suture was performed with a total blood loss of 498 g (Fig. 1B). However, the anemia and bleeding continued, and additional blood transfusion of RBCs and fresh-frozen plasma (FFP) was required (detailed treatments and clinical course are shown in Fig. 2). On POD 3, the amylase level of abdominal drainage fluid was 79 U/L. On POD 7, immunosuppressive treatment with prednisolone at 60 mg/day (1 mg/kg/day) was initiated at the discretion of a hematology specialist (M. F), based on the suspicion of AHA for the first time. On POD 8, she developed hemorrhagic shock, and CE-CT showed extravasation from the surface of the right posterior segment of the liver (Fig. 1C). She underwent emergent angiography with embolization of the right posterior hepatic artery (Fig. 1D) and was managed on a ventilator. A significant decrease in FVIII activity (< 1%) was detected, and bleeding that required blood transfusion continued. She received recombinant activated factor VII (rFVIIa) as a bypassing agent.

On POD 13, an inhibitor of FVIII was detected with the exclusion of von Willebrand factor and a lupus anticoagulant. She was, therefore, diagnosed with AHA. Immunosuppressive treatment with rituximab biweekly was added for uncontrollable hemorrhaging. She had received massive blood transfusion, administration of rFVIIa, and immunosuppressive therapy with prednisolone and rituximab. On POD 35, the inhibitor became undetectable, and the aPTT level gradually improved (Fig. 2). However, an infection of the intra-abdominal hematoma and concurrent fungal and cytomegalovirus infections due to immunosuppression developed. Along with the hemorrhagic tendency, thrombosis developed, resulting in multiple cerebral infarctions. Her general condition gradually worsened despite continuous treatment. Finally, she died of multiple organ failure on POD 71. The grades of postopancreatectomy hemorrhage (PPH) and postoperative pancreatic fistula (POPF) defined by the International Study Group of Pancreatic Surgery were as follows: PPH was grade C, and POPF was not identified [5, 6].

### Case 2 (At one and a half years after Case 1)

**Fig. 3:**
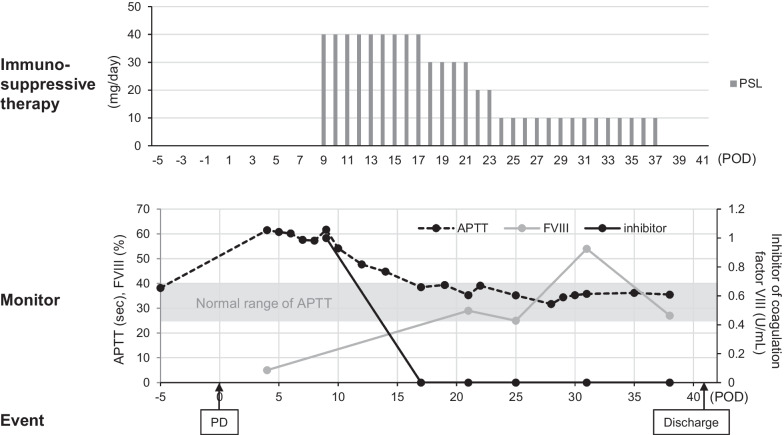
Detailed clinical course of Case 2. The top row shows the dose of immunosuppressive therapy (prednisolone). The second row shows the clinical course of aPTT, FVIII activity and the inhibitor of FVIII. The bottom row shows the clinical events. aPTT: activated partial thromboplastin time; FVIII: coagulation factor VIII; PD: pancreatoduodenectomy; POD: postoperative day; PSL: prednisolone

The patient was an 82-year-old man suffering from obstructive jaundice who was diagnosed with distal cholangiocarcinoma. The aPTT level at the time of the initial visit was 38.2 s. PD was performed with 577 g of blood loss and no blood transfusion. On POD 1, the amylase level of the abdominal drainage fluid was 8139 U/L. On POD 3, a small amount of blood via abdominal drainage was observed. The amylase level of the abdominal drainage fluid was 2932 U/L. On POD 4, his aPTT level was prolonged to 61.5 s, while his PT level was within the normal range. We suspected the possibility of coagulopathy, including AHA, and consulted a hematology specialist (M.F). No increase in hematogenous drainage or progression of anemia was observed. On POD 8, subcutaneous hemorrhaging of the median wound was observed. On POD 9, the FVIII activity was found to have decreased to 5%, and immunosuppressive treatment with prednisolone at a dose of 40 mg/day (1 mg/kg/day) was started based on the suspicion of AHA (detailed treatments and clinical course are shown in Fig. 3). On POD 15, an inhibitor of FVIII was detected, and he was diagnosed with AHA. On POD 17, the aPTT level had normalized, and an inhibitor of FVIII was undetectable with prednisolone. Subcutaneous hemorrhaging of the median wound, which could be controlled with gauze compression, was continued intermittently until normalization of the aPTT. Blood transfusion was not needed. No hemorrhagic complications other than subcutaneous hemorrhaging developed. Although he had pancreatic jejunal anastomotic leakage and pancreatic fistula after the initiation of prednisolone, these complications improved conservatively, and abdominal drains were removed on POD 33. The dose of prednisolone was gradually decreased, with prednisolone being withdrawn on POD 37. He was discharged on POD 41. PPH was grade A, and POPF was grade B [5, 6]. At 3 months after PD, his aPTT level was maintained within the normal range, and the inhibitor remained undetectable.

## Discussion

We encountered two cases of AHA in the perioperative period of PD. Case 1 showed a prolonged aPTT preoperatively; however, PD was performed as scheduled. Subsequently, postoperative hemorrhaging continued, and despite hemostatic treatment using rFVIIa and continued immunosuppressive treatment with corticosteroids and rituximab for AHA, the patient eventually died due to subsequent infections. Case 2 developed AHA postoperatively but responded well to corticosteroid treatment. Thus, although these two patients showed similar perioperative conditions during PD, they had markedly different outcomes. Our experience indicates that the recognition and early diagnosis of AHA are important for reducing mortality in the perioperative period of invasive surgery, such as PD.

Patients with AHA present with isolated prolonged aPTT with abnormal bleeding symptoms, such as subcutaneous, muscle, gastrointestinal, genitourinary, or retroperitoneal bleeding [1]. Although more than 50% of cases are idiopathic, AHA possibly develops as a paraneoplastic syndrome [1]. Malignancies were reported to account for 6–18% of the underlying disease in AHA [3, 7–9]. Among solid tumors, the incidence of prostate cancer, lung cancer and colon cancer was reported to be high, although there were a few reports of pancreatic cancer and bile duct cancer [10]. Therefore, patients with malignancies could develop AHA both before and after surgery. There are some case reports regarding the development of AHA during the preoperative or postoperative period, including cases involving PD [11–15]. It has also been suggested that AHA may be triggered by a surgical procedure or infections such as cholangitis [12, 15]. Although the mechanism underlying the development of AHA is still unclear, in addition to neoplastic syndrome, an inflammatory state (e.g., from surgery or infection) may trigger the development of AHA. Tumors in the pancreatic head, including pancreatic cancer and bile duct cancer, often lead to cholangitis, cholecystitis, and pancreatitis. Therefore, especially when patients with malignancy develop these inflammatory diseases preoperatively, a systemic examination for bleeding symptoms and reconfirmation of aPTT are desirable. Although postoperative hemorrhage after PD is often caused by POPF, the possibility of AHA should also be considered.

The treatment of AHA reportedly consists of four pillars: prevention of bleeding; treatment of the underlying disease; hemostatic treatment using an FVIII agent, desmopressin, and bypassing agents (rFVIIa or activated prothrombin complex); and immunosuppressive therapy (e.g., steroids, cyclophosphamide and rituximab) for eradication of the inhibitor [1]. To prevent bleeding, invasive procedures should be avoided if AHA develops in the perioperative period. Although treatment of the underlying disease may cure AHA, it has been suggested that surgery should be delayed until inhibitor eradication when feasible [16]. Therefore, when AHA occurs preoperatively, the suitability for surgery should be carefully decided. Regarding hemostatic treatment, the early diagnosis and initiation of treatment are crucial, as a delayed diagnosis was reported to be associated with a poor response or greater consumption of hemostatic agents [17, 18]. Furthermore, although not used in present cases, emicizumab, which is a monoclonal bispecific antibody that mimics the function of FVIII and has been reported to be efficacious for AHA in recent years [19], may be considered for future cases. Immunosuppressive therapy for eradication of the inhibitor may lead to adverse events, such as compromised immunity and delayed wound healing, so careful management is required for postoperative patients. Since the severity of bleeding was reported to be independent of the inhibitor titer and residual FVIII [1], these treatments should be tailored to the clinical symptoms.

In Case 1, the patient supposedly developed AHA at the time of acute cholecystitis before PD, as evidenced by the prolonged aPTT following cholecystitis. Retrospectively, possible causes of the severe clinical course of Case 1 include: PD being carried out as planned without consideration of the prolonged aPTT; the addition of second surgical invasion; the delayed diagnosis and commencement of treatment for AHA (duration from confirmation of the prolonged aPTT to the commencement of AHA treatment: 11 days); and secondary infection during prolonged treatment period due to uncontrollable hemorrhaging. PD should have been postponed, and the cause of prolonged aPTT should have been investigated in detail for Case 1. In Case 2, the patient developed AHA in the early postoperative period after PD. We suspected AHA in this patient due to minor hemorrhaging and prolonged aPTT, based on our experience with Case 1, and initiated early treatment for AHA (duration from confirmation of the prolonged aPTT to the commencement of AHA treatment: 4 days). As a result, hemorrhaging was controlled, and adverse events from steroids were minimal. Thus, the duration from the onset to the initiation of treatment may be crucial. If malignancies and inflammation from surgery or infection can serve as triggers for AHA development, oncological surgeons may encounter AHA more frequently than previously reported. Keeping AHA in mind during the perioperative period is thus important.

## Conclusion

AHA may be more common than previously reported. When unexplained prolonged aPTT or abnormal bleeding symptoms are detected, we should consult specialists in hematology as early as possible. The early diagnosis and management of AHA may help to reduce mortality.

## Data Availability

None.

## Abbreviations

AHA: Acquired hemophilia A
aPTT: Activated partial thromboplastin time
CE-CT: Contrast-enhanced computed tomography
FFP: Fresh frozen plasma
FVIII: Coagulation factor VIII
PD: Pancreatoduodenectomy
POD: Postoperative day
POPF: Postoperative pancreatic fistula
PPH: Postopancreatectomy hemorrhage
PSL: Prednisolone
PT: Prothrombin time
RBC: Red blood cell
rVIIa: Recombinant activated factor VII
